# Liquid-Crystal-Filled Side-hole Fiber for High-Sensitivity Temperature and Electric Field Measurement

**DOI:** 10.3390/mi10110761

**Published:** 2019-11-10

**Authors:** Yijian Huang, Ying Wang, Chun Mao, Jingru Wang, Han Wu, Changrui Liao, Yiping Wang

**Affiliations:** 1 Guangdong and Hong Kong Joint Research Centre for Optical Fibre Sensors, College of Physics and Optoelectronic Engineering, Shenzhen University, Shenzhen 518060, China; huangyijian@email.szu.edu.cn (Y.H.); 2172281502@email.szu.edu.cn (C.M.); wangjingru@email.szu.edu.cn (J.W.); wuhan2018@email.szu.edu.cn (H.W.); cliao@szu.edu.cn (C.L.);; 2 Guangdong Laboratory of Artificial Intelligence and Digital Economy (SZ), Shenzhen University, Shenzhen 518060, China; 3 Key Laboratory of Optoelectronic Devices and Systems of Ministry of Education and Guangdong Province, Shenzhen University, Shenzhen 518060, China

**Keywords:** side-hole fiber, liquid crystal, fiber optics sensors

## Abstract

We propose a highly sensitive sensor based on a nematic liquid-crystal-filled side-hole fiber. The liquid crystal is precisely filled into an air hole of the optical fiber using a method of manually gluing in the fusion splicer. Due to the coupling between the liquid crystal waveguide and the fiber core, multiple response dips appear in the transmission spectrum of the device. When an external temperature or electric field variation is applied to the liquid crystal and its refractive index changes, the transmission spectrum of this device will shift accordingly. Temperature and electric field response tests were performed on the device in the experiment, and the obtained temperature and electric field sensitivities were as high as −1.5 nm/°C and 3.88 nm/V_pp_, respectively. For the exhibited advantages of being easy to manufacture, low cost, and high sensitivity, the proposed sensor is very promising for actual application in temperature or weak electric field monitoring.

## 1. Introduction

Side-hole fiber (SHF) is a typical microstructure fiber with two air holes beside its core. Due to the novel internal configuration of this fiber, SHF has become a research hotspot in the field of fiber sensing in recent years. And many sensor structures based on SHF have been reported, such as long-period fiber grating [1], fiber Bragg grating [2], Sagnac interferometer [3], Fabry–Perot Interferometer [4,5], Mach–Zehnder Interferometer [6], and Michelson Interferometer [7]. However, the research on the use of SHF integrated electro-optical materials for the fabrication of all-fiber electro-optic functional devices has hardly been demonstrated.

In recent years, the application of liquid crystal in the display field has become ubiquitous in daily life, but in the non-display field, liquid crystal also has a very large potential application value [8,9,10,11]. The latest research progress on novel liquid crystal functional materials [12,13,14,15,16] makes it promising to be used in many fields such as smart textiles, energy-efficient electronics, and smart laser protection [17]. Liquid crystals have some unique physical properties, such as good fluidity at normal temperature, under the action of an external electric field, the direction of the liquid crystal molecules rearrange, resulting in a change in the refractive index of the liquid crystal, making it possible to design a liquid-crystal-based fiber electro-optic device by integration of liquid crystal material with optical fiber [18,19,20,21,22]. 

In this paper, we propose a directional coupling structure based on SHF and experimentally study the temperature- and electric-field-sensing characteristics of this structure. By filling the liquid crystal in an air hole of the SHF, a liquid multimode optical waveguide parallel to the fiber core is obtained. This waveguide supports a large number of guided modes. When the phase-matching condition is satisfied, the fundamental mode in the fiber core and the guided mode in the liquid waveguide are coupled at specific wavelengths, which results in many resonant dips in the transmission spectrum of the device, and the wavelengths of these response dips are highly sensitive to the refractive index of the liquid crystal. When the external temperature or electric field variation is applied to change the refractive index of the liquid crystal, the spectrum of the device will shift correspondingly, which allows this device to be used as a sensor. The highest temperature sensitivity obtained in the experiment is −1.5 nm/°C, and the sensitivity of the device to the external electric field is 3.38 nm/V_pp_.

## 2. Sensor Fabrication and Theoretical Analysis

**Figure 1:**
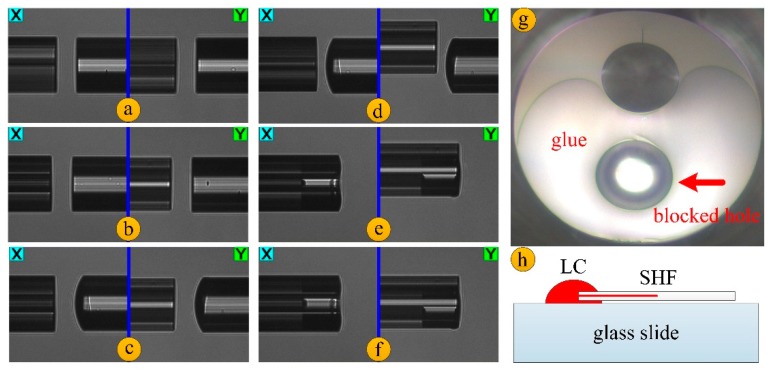
Schematic diagram of the device fabrication. (**a**–**f**) Flow chart of the fiber pre-processing. (**g**) End-face microscope image of the side-hole fiber (SHF) after gluing. (**h**) Liquid crystal filling with capillary action.

The manufacturing process of the sensor is shown in Figure 1. In the experiment, we used an SHF with two air holes on each side of the fiber core. The cladding diameter of the SHF matches the cladding diameter of the standard single-mode fiber. The diameter of the air hole in the SHF is ~35 um, and the distance between the center of the air hole and the center of the fiber core is ~24 um. In order to selectively fill the liquid crystal into an air hole of the SHF, a method of manually gluing assisted by the fusion splicer (FSM-60s, Fujikura, Tokyo, Japan) was used, as illustrated in Figure 1a–f. First, we put the single-mode fiber and the SHF into the fusion splicer, respectively, and manually drove the motor of the fusion splicer to move the fiber to the center of the field of view. At this time, we can see that the direction of the two holes on the left side of the SHF is arbitrary (Figure 1a). After that, we manually rotated the fiber on the left side, until the arrangement of the two air holes was parallel to the X or Y direction, so that when we observe the SHF in the fusion splicer, the screenage of the two air holes should be overlapping in the x field of view and separate in the y field of view, as shown in Figure 1b. Then we glued the right single-mode fiber to a small amount of UV glue and put it into the fusion splicer again (Figure 1c). We moved the left SHF by manual motor drive mode, and then the left fiber was misaligned by a certain distance to ensure that the right fiber was only aligned with one air hole on the left SHF. We pushed the fiber on the right side forward, applied the glue to an air hole, and then quickly removed the fiber on the right side to ensure that another air hole was not blocked by the glue due to excess glue on the cross section of the SHF (Figure 1d,e). Finally, the curing operation of the glue was performed, and Figure 1f shows the fiber image after the glue had cured. Figure 1g is a microscopic image of the SHF facet after being glued. It is obvious that one air hole is blocked by the glue and the other air hole is kept open, so that the liquid crystal can be easily filled in the unblocked air hole of the SHF by capillary effect. In the experiment, the pretreated SHF was inserted into the liquid crystal droplets on the glass slide. Due to the capillary action and the low viscosity of the liquid crystal, a liquid-crystal-filled SHF of several centimeters in length can be obtained in a short time.

**Figure 2:**
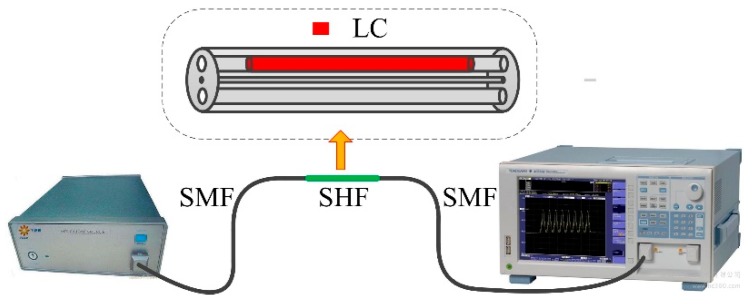
Schematic diagram of the experimental test system. Inset: structure diagram of liquid-crystal-filled SHF.

Our experimental test setup is shown in Figure 2. We spliced a standard single-mode fiber at each end of the liquid-crystal-filled SHF. In order to reduce the splice loss, a negative pressure was applied on the other end of the SHF to move the liquid crystal rod inside to the fiber for a few millimeters before the fiber splicing, so that the liquid crystal in the fiber will not be carbonized during the discharge fusion. A broadband ASE light source and an optical spectrum analyzer (OSA, AQ7360C, Yokogawa, Tokyo, Japan) were employed to test the transmission spectrum of the sample.

**Figure 3:**
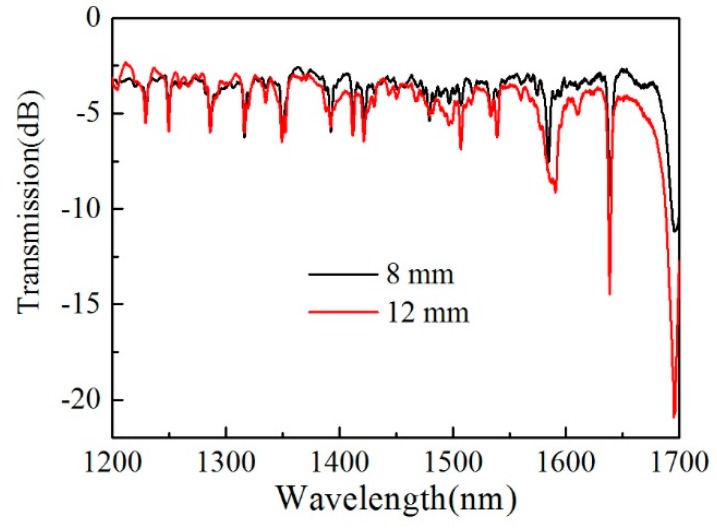
Spectra of 8 mm (black curve) and 12 mm (red curve) samples, respectively.

The liquid crystal filled in the SHF is a typical nematic liquid crystal (E7, n_o_ = 1.517 , n_e_ = 1.741 at 589.3 nm and 20 °C, Suzhou King Optronics Co., Ltd., Suzhou, China), the large diameter of the air hole running along the fiber core and high refractive index difference between the liquid crystal and the background silica material make the liquid rod being a multimode waveguide. When the phase-matching condition is satisfied, the coupling between the fundamental mode in the fiber core and the guided modes in the liquid waveguide will occur, resulting in light coupling into the liquid waveguide at the phase-matching (resonant) wavelengths. Figure 3 shows the transmission spectra of two samples with liquid rod lengths of 8 mm and 12 mm, respectively. We can see that there are multiple resonant dips in the spectra of the two samples, and the resonant wavelengths of the two samples are nearly the same, except for the extinction ratios. The higher extinction ratio of the 12 mm sample may be benefit from its longer length, which increases the coupling strength of the device compared with that of the 8 mm sample.

**Figure 4:**
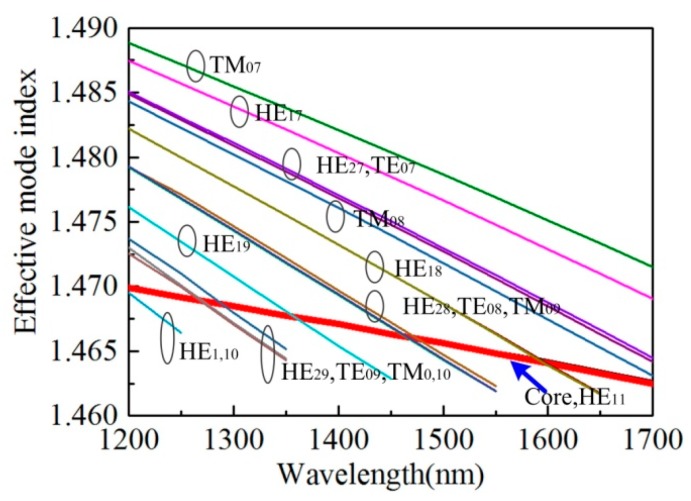
Calculated dispersion curves of several liquid crystal waveguide modes and fiber core fundamental mode.

To illustrate the operation principle of the proposed device, dispersion curves of the fundamental core mode and several liquid crystal waveguide-guided modes have been calculated through finite element method (FEM), as shown in Figure 4. In the FEM model, fiber geometric parameters and material refractive indices are in accordance with aforementioned values. And Figure 4 only shows dispersion curves of several orders of transverse electric (TE), transverse magnetic (TM), and hybrid electromagnetic (HE) modes supported by the liquid crystal waveguide for clarity. The red thick curve indicates the dispersion curve of the fundamental core mode (Core HE_11_). The intersections of the core mode curve and liquid crystal waveguide mode curves indicate the phase-matching conditions, where light propagated in the fiber core mode can be coupled into the liquid crystal waveguide, thus resulting in resonant dips in the device transmission spectrum. Note that the resonant wavelengths of the simulation results do not coincide exactly with that of the experiment, as shown in Figure 3. This is mainly due to the inaccuracy of geometry parameters and materials’ refractive indices used in our simulation, compared with that of the experiment, since slight difference in material refractive index or fiber structure parameters will result in an apparent shift in resonant wavelengths [23]. According to Figure 4, all of the dispersion curves of the liquid crystal waveguide will move upward once the refractive index of liquid crystal becomes larger, and at the same time, the intersections of fiber core mode and liquid crystal waveguide mode will move towards longer wavelengths, implying red shift of the resonant wavelengths of the device, and vice versa. When temperature increases, the resonant wavelength of the device will show blue shift, since the liquid crystal used here exhibits a negative thermal-optic coefficient [24]. Meanwhile, the thermo-optic coefficient of silica is about two orders of magnitude smaller than that of liquid crystal and thus, can be ignored. When voltage is applied to the device, the refractive index of liquid crystal will increase due to molecular realignment, hence the resonant wavelengths will shift to longer wavelengths. 

## 3. Sensing Experiments 

**Figure 5:**
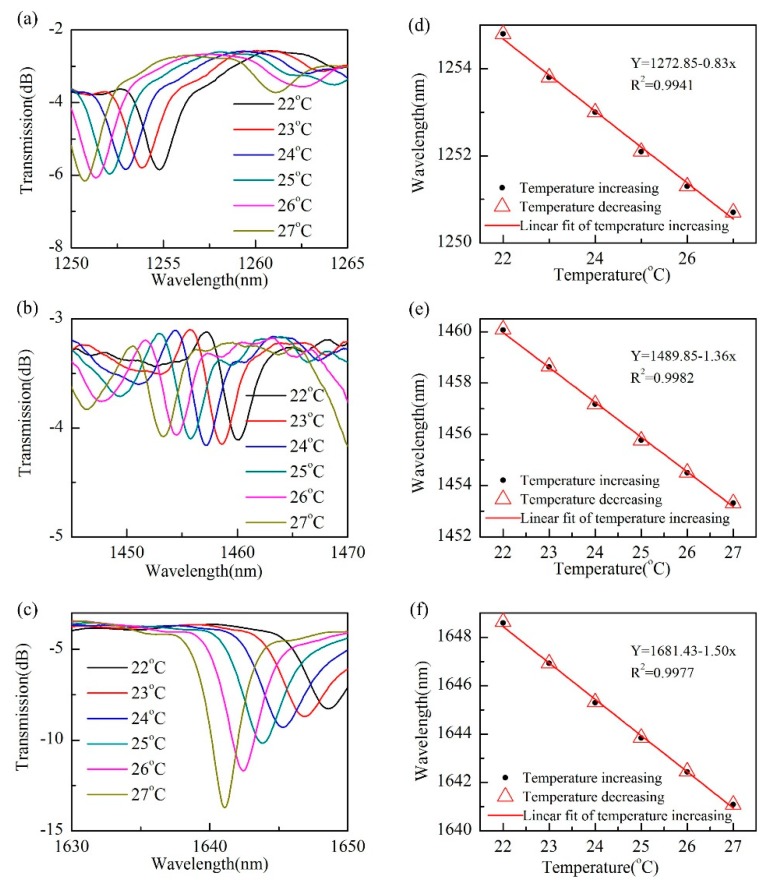
Temperature response of the sensor. (**a**–**c**) Transmission spectra of the sample under different applied temperature; (**d**–**f**) dip wavelength as a function of the applied temperature.

In order to study the response of the device to temperature changes, the sample with length of 12 mm was placed in a temperature oven (LCO 102, Ecom, Chrastany, Czech Republic), which can increase the temperature from room temperature to 100 °C with an accuracy of 0.1 °C. Here, we increased the temperature from 22 °C to 27 °C, and recorded the transmission spectrum of the sample at each temperature. To ensure the accuracy of the experimental data, the experimental data were recorded about 15 min after the temperature stabilized. The experimental results showed that as the temperature increased, the wavelength of the response dips in the transmission spectrum of the sample shifted to the short-wavelength direction, which is blue-shifted. This is mainly due to the decrease in the refractive index of the liquid crystal when the temperature rises, so that the dispersion curve of the liquid crystal waveguide modes moves downward, and the intersection in Figure 4 will shift to shorter wavelengths. Figure 5a–c shows the spectral variation at three different wavelengths with increasing temperature, respectively. It can be seen that as the temperature increased, the spectrum shifted to the short-wavelength direction very regularly. In order to verify whether the sample has a good recoverability, the sample was tested for temperature rise and temperature drop. Figure 5d–f shows the relationship between the resonant dips wavelength and temperature value when temperature increased and temperature decreased, respectively. It can be seen that the wavelength positions of the resonant dips during the temperature increasing and decreasing process are well coincident. At the same time, we linearly fitted the data during the temperature-increasing process. We can see that our sensor has a good temperature linear response. The temperature sensitivity of these three response dips is −0.83 nm/°C, −1.36 nm/°C, and −1.50 nm/°C, respectively.

**Figure 6:**
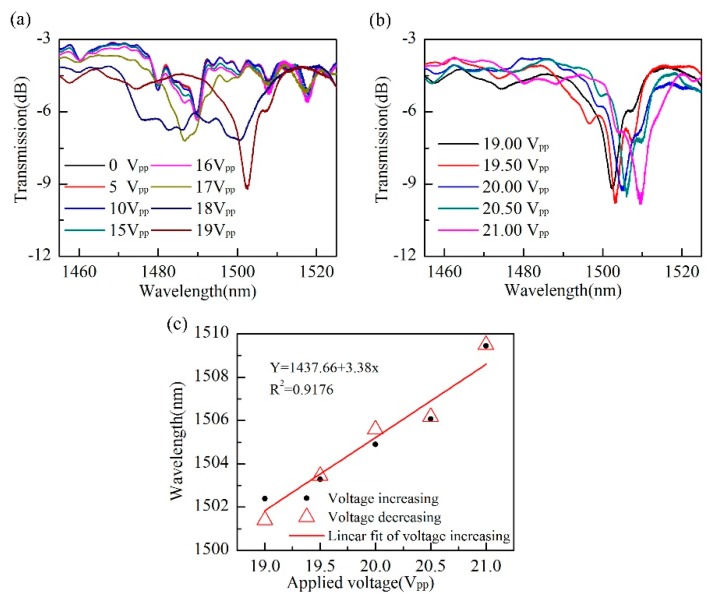
The response of this sensor to the variation of external electric field. (**a**,**b**) Transmission spectra of the sample under different applied electrical voltages; (**c**) dip wavelength as a function of the applied electric voltage.

We placed the liquid-filled SHF in the middle of two conductive glasses and separated the two glasses with some single-mode fibers without coating, ensuring a 125 μm spacing between the two conductive glass plates. Before electric field is applied, the optical transmission characteristics of the liquid crystal waveguide are mainly determined by n_o_. When a voltage is applied to the glass, a spatial electric field will be formed within the two glass plates. Under the action of the electric field, the liquid crystal molecules in the SHF will overcome the anchoring energy, and the long axis of the liquid crystal molecules will gradually rotate toward the electric field direction. The n_e_ start plays an important role, and the effective refractive index of the liquid crystal waveguide increases. Thereby, affecting the transmission spectrum of the sample and resulting in a red shift of the dips. Apply a voltage to the conductive glass and change the intensity to observe the variation in the transmission spectrum of the sample. Here we used 1 kHz of sinusoidal electrical signal. Figure 6a,b show the response of the sample transmission spectrum as the applied voltage increased. When the voltage is lower than 15 V_pp_, we can see that the transmission spectrum has not changed significantly, this may be because the applied electric field was too weak to realign the liquid crystal molecules. And the resonant dip begins to shift irregularly between 15 V_pp_ and 19 V_pp_. This may be the electric field effect just overcomes the liquid crystal anchoring energy, resulting in the irregular change of refractive index of liquid crystal under the action of thermal motion of liquid crystal molecules and external electric field. At 19 V_pp_ to 21 V_pp_, the alignment of the liquid crystal molecules gradually starts, from the initial escaped radial structure, to parallel the direction of the electric field [25], which leads to an approximately linear change in the refractive index of the liquid crystal, and finally, causes the resonant dip in the transmission spectrum to approach a linear shift, as shown in Figure 6b. We performed a voltage increasing and voltage decreasing test on the sample to verify that the electric field response of the sensor is repeatable. Figure 6c shows the relationship between the resonant wavelengths and voltage when voltage is increasing and when voltage is decreasing. This clearly shows that during the voltage increasing and decreasing process, the response dip wavelengths of the sample are basically coincident, which indicates that our samples can be repeatedly used in practical applications. A linear fit of the resonant wavelength shift versus voltage increasing indicates that our device has an electric field sensitivity up to 3.38 nm/V_pp_ (9.56 nm/V_rms_). This is mainly benefited from the high sensitivity of waveguide coupling structure used here. The obtained sensitivity is higher than that of previously reported liquid-crystal-based fiber structures, such as interferometers (0.53 nm/V_rms_) [18], whispering-gallery-mode microresonator (0.01 nm/V_rms_) [25], fully filled (~1.8 nm/V_rms_) [26], and selectively filled photonic crystal fibers (5.594 nm/V_rms_) [22]. Meanwhile, our device exhibits lower measurement threshold for electric field sensing.

## 4. Conclusions

A high-sensitivity sensor based on liquid-crystal-filled SHF is proposed, which has the advantages of a simple structure, being easy to fabricate, and having low cost. This device can be used for high-sensitivity temperature or electric field sensing. Experimental results show that the device can realize low-voltage electric field sensing. The temperature and electric field sensitivity of the device are −1.50 nm/°C and 3.38 nm/V_pp_, respectively, and the device response to external temperature or electric field has good repeatability, these significant advantages make it promising for sensing applications.
